# Post-operative rehabilitation following traumatic anterior shoulder dislocation: A systematic scoping review

**DOI:** 10.1177/17585732221089636

**Published:** 2022-03-31

**Authors:** Martha Coyle, Anju Jaggi, Lewis Weatherburn, Helena DanielI, Rachel Chester

**Affiliations:** 1 School of Health Sciences, Faculty of Medicine and Health Sciences, University of East Anglia, Norwich, UK; 2 Physiotherapy Department, 7597 Royal National Orthopaedic Hospital NHS Trust, Stanmore, UK; 3 Physiotherapy Department, 156671 Norfolk and Norwich University Hospital NHS Trust, Norwich, UK

**Keywords:** Arthroscopic surgery, physical therapy modalities, review, shoulder, trauma, instability

## Abstract

**Background:**

This systematic scoping review aimed to describe the content of post-operative rehabilitation programmes, and outcome measures selection following stabilisation surgery for traumatic anterior shoulder dislocation (TASD).

**Methods:**

An electronic search of Medline, EMBASE, CINAHL and AMED was conducted (2000–2021). Any cohort or clinical trial of patients receiving post-operative TASD rehabilitation were included. Study selection, data extraction and quality appraisal were undertaken by two independent reviewers.

**Results:**

Twelve studies including fourteen treatment programmes were eligible. Period of post-operative immobilisation ranged from 1 day to 6 weeks, with exercise introduced between 1 and 7 weeks. Strengthening exercises were introduced between 1 and 12 weeks. Two studies described “accelerated” rehabilitation programmes, differing in immobilisation period and exercise milestones. No increased recurrence was reported in professional footballers. Two studies compared rehabilitation programmes, one not randomised, the other 18 years old. There was variability in selected outcomes measures, with only 4 studies using a common measure.

**Discussion:**

There is minimal evidence to guide post-operative rehabilitation, variability in immobilisation periods and when exercise is introduced. There is no consensus on the definition of accelerated rehabilitation, or outcome measure selection. Clinical consensus of standardised terminology and stages of rehabilitation is required prior to efficacy studies.

## Introduction

Traumatic anterior shoulder dislocations (TASD) account for between 80% and 90% of shoulder dislocations^1–5^ and are the most common dislocation seen in accident and emergency and trauma clinics.^2,5^ TASD occurs when the humeral head is forced anteriorly out of the glenoid fossa after an external blow to the arm, typically when the shoulder is abducted and externally rotated.^6^ Although this type of injury can occur at any age, peak incidence occurs in males aged 10–40 typically involved in contact sports,^5,7,8^ with a second smaller peak in elderly people who sustain the injury during low-level falls.^2,9,10^TASD can result in structural problems such as a Bankart lesion (avulsion of the inferior glenohumeral ligaments from the anteroinferior labarum) and a Hills-Sachs lesion (a compression fracture of the posterosuperolateral humeral head),^11,12^ both of which predispose to chronic post-traumatic instability,^13^ with recurrent dislocation rates as high as 85%–92% in the young sporting population.^14^

Over half of people managed conservatively will experience a recurrent dislocation.^13^ There has therefore been a shift to immediate surgical intervention in an attempt to lower rates of recurrent dislocation.^15^ Arthroscopic Bankart repair, is currently the most commonly performed surgery for anterior shoulder instability.^16,17^ The Laterjet is gaining increasing popularity in the presence of osseous glenoid defects, particularly in the contact athlete.^18,19^

Reports of surgical advancements are rarely supplemented by alterations in post-operative rehabilitation programmes. The content of rehabilitation, if described at all, is rarely in enough detail to replicate. Studies comparing different rehabilitation programmes, irrespective of publication date, are few.^20,21^ Accelerated rehabilitation programmes, with minimal post-operative immobilisation, have been documented as early as 2003^20^ and have gained increasing profile following a later publication in 2016.^22^ However, it is unclear how the individual components of accelerated rehabilitation differ from more traditional rehabilitation programmes. A lack of formal comparison between rehabilitation programmes guidelines is confusing for clinicians and researchers wishing to identify “standard practice”. There is currently no synthesis of the content or outcome measures documented in research studies describing post-operative rehabilitation for TASD.

The objective of this systematic scoping review is to describe the rehabilitation programmes and outcome measures described in the primary literature following stabilisation surgery for TASD, the secondary aim being where possible, to compare the effectiveness of these programmes.

## Methodology

This review was conducted according to the guidelines for the preferred reporting items for systematic reviews and meta-analyses (PRISMA) statement for scoping reviews^23^ and has been registered on the International prospective register of systematic reviews (PROSPERO) (Registration number CRD42020201438).

### Data sources and search

MEDLINE, EMBASE (via Ovid) and CINAHL were searched from January 2000 to January 2020 using the NHS electronic library. The full Medline search strategy is presented in supplementary file 1 with search terms adapted for other databases. No language limits were applied. Reference lists of eligible publications were hand searched.

### Study selection and eligibility criteria

Two independent reviewers (MC and LW) screened all retrieved titles and abstracts against pre-defined eligibility criteria. Full texts of potentially eligible papers were then sourced and reviewed (MC and LW).

Any study published in a peer reviewed journal from January 2000 that met the following criteria were included in the review: Participants, irrespective of age, who had undergone any type of anterior shoulder stabilisation surgery following at least one TASD identified as such by any professional healthcare practitioner or diagnostic imaging. Interventions must have been delivered by or involve one or more physiotherapists.

Studies were excluded from the review if they included participants with atraumatic shoulder instability or who had undergone surgery for degenerative superior labral anterior posterior lesions (SLAPs), posterior or multidirectional instability. The following interventions were excluded: treatment provided over the telephone, in which there had been no face to face contact with a physiotherapist or where ice/hot packs were the only form of physiotherapy.

### Data extraction, categorisation and critical appraisal

Two independent reviewers (MC and LW) extracted data and entered it onto a custom designed data extraction form (Supplementary file 2). The form was piloted tested by MC and LW on three studies^21,22,24^ and refined in response to any ambiguities. Details of any immobilisation period post operatively were recorded, and each treatment and exercise intervention were categorised for ease of synthesis and presentation of results. Exercise categories were developed and defined during consensus meetings including all the review authors and included: Range of Motion (ROM), Isometrics, Strength, Movement Re-education, Stability and Proprioceptive/Neuromuscular Training. Clinical trials comparing rehabilitation programmes were appraised using the Physiotherapy Evidence Database (PEDro) scale for randomised controlled trials.

## Results

### Study selection

**Figure 1.**
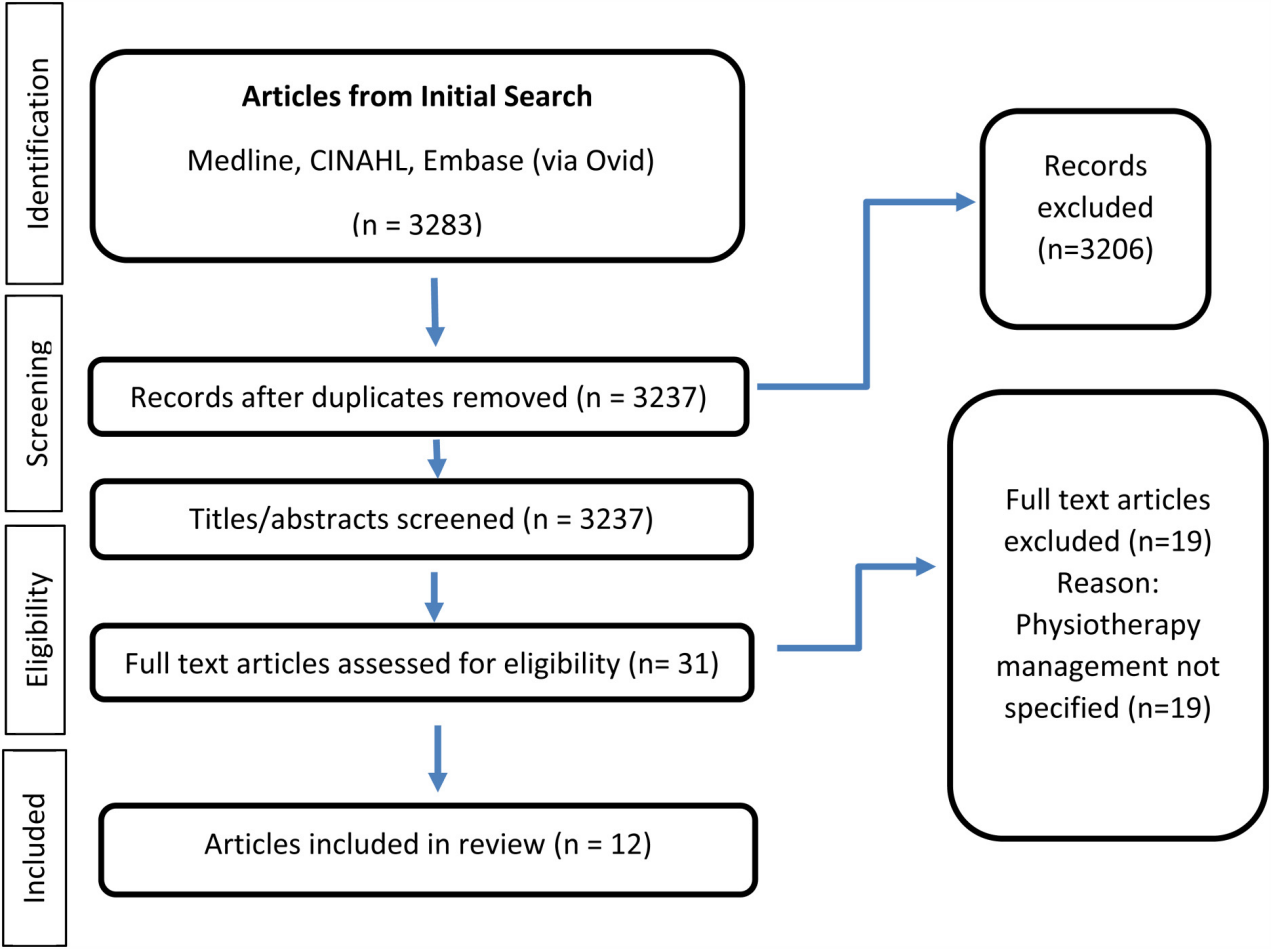
PRISMA flow chart outlining the literature search and study selection.

The search strategy yielded 3283 citations, of which 46 were removed due to duplication. Following title and abstract screening 31 were selected for a full text review. A further 19 were excluded as the physiotherapy management was not specified. 12 studies were finally included in the scoping review. A PRISMA summary is presented in Figure 1.

### Study characteristics

In total, information from 14 treatment programmes were extracted from 12 papers covering 12 studies, 5 of which were clinical trials^20,21,25–27^ and 7 of which were observational studies.^22,24,28–32^ Of the 5 clinical trials in this review, two studies compared 2 groups receiving different rehabilitation programmes following surgery after TASD.^20,21^ For the remaining 3 studies;^25–27^ the aim was to compare either different surgical interventions, in which both groups received the same physiotherapy, or surgery versus physiotherapy. Two studies describe accelerated rehabilitation programmes, one of which was a cohort study^22^ and one a clinical trial.^20^ The total number of participants included 659 males and 150 females with an age range of 15–57 years. Six papers (45% of participants) state that participants sustained a TASD due to a sport or physical activity. Full participant demographics are outlined in supplementary file 3.

### Treatment programmes

The primary objective for 4 of the papers in this review was to describe the treatment programmes delivered by physiotherapists. For the remaining 8 papers, for whom this was not an objective, only a brief or general outline of treatment programmes were provided.

The exercises in each treatment programme were taken verbatim from the papers, categorised and summarised for clarity and conciseness in supplementary file 4. Exercises were performed independently by the patient, unless indicated otherwise.

### Immobilisation

All but one of the studies^20^ described an immobilisation period (See Table 1). This ranged 2 to 6 weeks. 11 of the 14 programmes used a sling, and 3 studies an alternative shoulder immobiliser.^27,28,30^ Five programmes included a period of absolute shoulder immobilisation in a sling,^20,21,24,26,31^ of which 2 explicitly recommended continued use of the hand, wrist, and elbow.^20,21^ The remaining 9 programmes all permitted some form of passive or active assisted shoulder movement during the immobilisation period.

**Table 1.** Summary of immobilisation period and intervention milestones.

	**Immobilisation Period**					**Treatments (Week commenced)**				
**Reference**	Yes/No	Duration	Position	Device	Permitted movements	PROM/ AAROM	AROM	Isometric exercises	Strength	Restricted Movements
**Bottoni, 2002 USA**	Yes	4 weeks	N/S	Sling	PROM and AAROM movements Isometric contractions Limited AROM	Week 1	Week 4	Week 1	Week 4	N/S
**Damkjaer, 2015 Denmark** **(standard care group)**	Yes	6 weeks	N/S	Sling	PROM and AAROM of wrist and fingers PROM and AAROM ER to neutral	Week 1	Week 4–6 (at surgeon's discretion)	N/S	Week 6	N/S
**Damkjaer 2015, Denmark** **(guideline group)**	Yes	0–3 weeks (depending on surgeon's discretion)	N/S	Sling	No movement permitted	Week 3	Week 6	Week 3	Week 6	0–6 weeks: Passive ER past 90° contraindicated 6–12 weeks: avoid stretching beyond goals, heavy lifting, plyometrics, push ups, military press. Avoid triceps dips, lat pull down or bar behind head until 16 weeks
**Dickens, 2017 USA**	Yes	6 weeks	N/S	Sling	No movement permitted	N/S	Week 6	N/S	Week 12	N/S
**Edmonds, 2003** **Canada**	Yes	3 weeks	N/S	Sling	No movement permitted	Week 4	Week 7	Week 7	Week 9	ER limited to 20° past neutral weeks 0–6. ER limited to 45° past neutral weeks 7–8
**Edwin, 2018 UK**	Yes	4 weeks	N/S	Sling	Allowing ER to neutral	Week 4	Week 4	N/S	N/S	N/S
**Eren, 2019 Turkey**	Yes	4 weeks	N/S	Sling	No shoulder ROM permitted. Tabletop activities without sling encouraged. Postural exercises with sling and isometric exercises for deltoid strengthening started the day after surgery	Week 5	Week 5	Week 4	Week 5	ER over 60° abduction not permitted in first 9 weeks
**Gibson, 2016 UK**	No	N/A	N/A	Sling (2–3 weeks)	PROM and AROM exercises within "safe zone”. CKC, isometric and through range recruitment exercises started in first week post-op. Use of hand in sling (light activities) encouraged.	Week 1	Week 1	Week 1	Week 1	0–4 weeks: No combined abd and ER. No forced end range mobilisation. 3–10 weeks: Avoid passive stretching into combined abd/ER
**Hiemstra, 2007 USA**	Yes	2–4 weeks	N/S	Shoulder immobiliser	At 2 weeks, PROM and AAROM exercises were for elevation and ER to neutral	Week 2	Week 6	N/S	Week 6	N/S
**Lutzner, 2009** **Germany**	Yes	6 weeks	N/S	Gilchrist cast	PROM 2x per day (anteversion up to 90°)	Week 1	Week 7	N/S	N/S	0–6 weeks: No abd/ER permitted
**Milchteim, 2016 USA**	Yes	6 weeks	N/S	Shoulder immobili-ser	Passive and gentle active assistive ROM exercises	Week 1	Week 5	N/S	Week 7	0–6 weeks: no active ER, extension, or abd allowed. Where a SLAP was repaired, no isolated biceps contractions allowed until week 5
**Ozturk, 2013 USA**	Yes	4 weeks	N/S	Sling	PROM in the scapular plane and pendulum exercises started in 1st week	Week 1	Week 3	N/S	Week 6	N/S
**Kim, 2003 South Korea** **(conventional group)**	Yes	3 weeks	Sling allowed the shoulder in 20° of abd and 40° of IR	Sling and pillow spacer	During this period, the patients allowed to flex their elbows and wrists and to wash the axilla daily	Week 3	N/S	N/S	Week 4	
**Kim, 2003 South Korea** **(accelerated group)**	No	N/A	N/A	Sling only when sleeping	Pendulum and sub maximal isometric exercises from day 1 post-op	Week 1	N/S	Week 1	Week 2	0–2 weeks: Forward elevation only to 90°

### Accelerated programmes

The two accelerated programmes^20,22^ began exercises 1–2 days post operatively, only using a sling when sleeping. Both programmes introduced shoulder mobilisation and strengthening exercises at different stages, the intensity being higher for Gibson's^22^ compared to Kim's^20^ programme. Gibson permitted some active movements from the second day post operatively whereas Kim only permitted pendular exercises and submaximal isometric exercises for the first week post operatively.

### Range of motion

Range of Motion (ROM) exercises were defined as any active, active assisted or passive movements for which the aim was to regain full range of shoulder movement. They were included in all programmes as one of the first interventions. Active assisted and passive movements were initiated in 7 programmes within the first week, one study did not report when they were commenced^31^ and the remaining 6 introduced them between the 2nd and 5th week. Active movement exercises were initiated between 1–7 weeks, the earliest being day 2 in Gibson's^22^ accelerated programme. Neither of Kim's^20^ protocols (conventional or accelerated) specified when active movements were introduced. Early exercises typically included pendulum exercises or using a rope and pulley.

### Isometrics

Isometrics were defined as muscle contractions where the length of the muscle and the angle of the joint do not change. These would often be completed early in the programme, and prior to active range of movement exercises in order to activate the shoulder muscles and restore some strength. The use of isometric exercises specifically for deltoid strengthening within the immobilisation period was reported in 5 of the 14 programmes.

### Strength

Strengthening exercises were defined where there was resisted movement through range with increasing load. These were distinct from isometric exercises. Strengthening exercises began between 1 and 12 weeks. Detailed descriptions of strengthening exercises and biweekly progressions were described in 3 programmes, including both accelerated programmes.^20,22^ Both Gibson and Kim begin strengthening in week one and week two respectively, although the intensity is higher in Gibson's^22^ than Kim's^21^ the latter being more comparable to that of Damkjaer's^24^ standard rehabilitation group or Ozturk's^29^ programme. These three papers describe similar exercises using dumbbells for the rotator cuff and scapular stabilisers from week 6. In addition to through range recruitment exercises, Gibson et al.^22^ also includes global strengthening work, incorporating closed kinetic chain exercises from week 1 and working the uninjured arm at high maximum voluntary contractions at week 3. It is not specified if these are isometric. Damkjær et al., despite not being defined as an accelerated programme, reports more advanced strengthening exercises (biceps, triceps, shrugs, rows, overhead dumbbell press, push ups) from week 6.^24^

Two studies^28,32^ did not specify whether or not strengthening exercises were used in their treatment programmes. The remaining 9 programmes provided guidelines on local scapula and rotator cuff exercises in different planes of motion. The use of increasing resistance bands and dumbbells were common progressions across these programmes.

### Movement Re-education

Movement Re-education was defined as any movement or exercise for which the aim was to restore functional movement patterns at the shoulder. It was reported in 3 programmes.^20,22,26^ Gibson et al.^22^ includes movement re-education in the form of proprioception and movement facilitation and *“through range activation incorporating the kinetic chain”* in the first week post operatively, Edmond^26^ introduces scapular retractions week 4 post operatively and Kim^20^ core exercises from week 10 post operatively.

### Stability

Stability exercises were defined as the application of fluctuating resistance loads incorporating the kinetic chain, while the patient stabilises the shoulder in a symptom-free position. Stability exercises were specified in 5 treatment programmes. Stabilisation exercises were a key focus in Gibson's^22^ treatment programme. They were initiated early in the programme and at a higher intensity compared to the other 4 programmes. The accelerated programme had patients recruiting dynamic stabilisers from 0–4 weeks, then completing *“preparatory and reactive stabilisation drills in risk positions”* whilst incorporating the kinetic chain beginning from 3 weeks post operatively. Damkjaer et al.^24^ reports the commencement of stability exercises 6 weeks post operatively in which the scapular stabilisers and rotator cuff are worked at high repetitions and low resistance. Stability exercises are commenced at week 10 in Kim's^20^ programme including ‘tubing exercises in 90/90 position’ and trunk strengthening. Eren et al.^21^ reports the use of closed kinetic chain exercises such as push ups and rowing from week 13 post operatively with the aim of strengthening the scapula stabilisers.

### Proprioceptive/neuromuscular training

Proprioceptive/Neuromuscular Training (PNMT) was defined as exercises or drills that train the nerves and muscles within the shoulder complex to react and communicate and included any sports specific or functional training, plyometrics, power, endurance, and advanced strengthening work. This was included in 6 programmes, 5 of which introduced in between 7 and 13 weeks post operatively. The remaining programme,^22^ focussed on professional footballers, commenced PNMT in the first week, initially in the form of maintaining cardiovascular fitness on a bike or incline treadmill, although this could be argued as general cardiovascular fitness than specific PNMT for the shoulder. At 3 weeks this progressed to completing non-contact drills and at 6 weeks incorporated function specific plyometrics, strength and endurance exercises, and “controlled falling drills”.

Four programmes provide details about advanced strengthening, power and endurance exercises^21,22,24,30^ and four include plyometric exercises.^21,22,24,29^

### Clinical outcome measures

Nine papers used 7 different patient reported outcome measures (PROMS) (See Table 2). The American Shoulder and Elbow Surgeon's Shoulder Score (ASES) was the most commonly used (4/12 papers), although this does include a clinician rated components. Otherwise at most, only 2 studies reported on any one PROM. The most reported outcomes were range of movement (4 papers), return to play, and the Rowe Score for Instability.^20,21,28,30^ Three papers report recurrence rates.^20,22,30^

**Table 2.** Summary of outcome measures.

**Author, Date, Country**	**Patient Reported Outcome Measure**							**Return to Play**	**Recurrence Rate**	**Range of Motion**	**Strength**	**Constant-Murley Score**	**Rowe Score**	**Other**
	**SANE**	**PSFS**	**WOSI**	**OISS**	**DASH**	**ASES**	**VAS**							
**Bottoni, 2002 USA**	✓							✓						
**Damkjaer, 2015** **Denmark (standard care group)**		✓	✓					✓		✓				
**Damkjaer 2015,** **Denmark (guideline group)**		✓	✓					✓						
**Dickens, 2017 USA**								✓						
**Edmonds, 2003** **Canada**														✓
**Edwin, 2018 UK**				✓										
**Eren, 2019 Turkey**					✓							✓	✓	
**Gibson, 2016 UK**								✓	✓					
**Hiemstra, 2007 USA**						✓				✓	✓			
**Lutzner, 2009** **Germany**										✓			✓	✓
**Milchteim, 2016 USA**						✓			✓	✓			✓	✓
**Ozturk, 2013 USA**						✓	✓			✓				
**Kim, 2003** **South Korea** **(conventional group)**						✓	✓		✓				✓	✓
**Kim, 2003** **South Korea** **(accelerated group)**						✓	✓		✓				✓	✓

Of the 5 clinical trials in this review, two compared 2 groups receiving different post-operative rehabilitation programmes. See supplementary file 5 for PEDRO scores.

Kim et al.,^20^ compared “accelerated” versus “conventional” rehabilitation. Participants were blinded to which group was accelerated. Participants receiving accelerated rehabilitation only wore their sling at night. There were no significant differences in outcome between groups at long term follow up in terms of reoccurrence, ROWE, ASES or return to activity. However, participants in the accelerated programme reported significantly less pain, at 6 weeks, were faster to regain full range external rotation and return to previous activities. Most of the patients in accelerated group were satisfied with early mobilisation and most of the conventional group were dissatisfied with brace immobilisation. Eren et al.^21^ scored poorly on the PEDro scale in part due to the absence of randomisation; participants could choose their treatment group. The trial consisted of a home-based group and supervised group. There were no significant differences in DASH, Rowe or Constant scores between groups at 6 or 12 months.

## Discussion

The primary objective of this scoping review was to describe the rehabilitation programmes and outcome measures described in the primary literature following stabilisation surgery for TASD. Our findings indicate that there is significant variability in rehabilitation milestones. The period of shoulder immobilisation ranged from 1 day to 6 weeks post-operatively, during which some studies advocated absolute immobilisation, whilst others allowed various amounts of active, active assisted or passive movement. Whilst all studies included these movements as one of their first interventions, the point at which they were introduced varied between one and seven weeks. Isometric exercises, when described, were introduced early, often before active movements in order to activate the shoulder muscles and restore strength. The introduction of strengthening exercises ranged between 1 and 12 weeks. Movement re-education for shoulder function and stability, and exercises incorporating the wider kinetic chain were reported in 3 and 5 studies respectively and were introduced between 1 and 10 weeks post-operatively. Proprioceptive, neuromuscular or cardiovascular training, sports or function specific, including plyometric, power and endurance training were included in 6 studies and were introduced between weeks 1 and 13. In summary, whilst there are similarities in the overarching components of rehabilitation programmes, the period of immobilisation, type, intensity and point at which specific exercises are introduced varied considerably.

A wide variation of outcome measures was reported with no more than 4 studies reporting the same outcome. Most studies did not include PROMs specifically designed for shoulder instability. The definition of return to play varied between studies, from getting back to playing sport^22,25,29^ or completing a full season without re-dislocation.^30,31^

The risks and benefits of early versus standard immobilisation versus are still inconclusive due to lack of research. The very limited evidence available from the two studies^20,22^ in our review suggests that early mobilisation may not be associated with increased instability. Early post-operative mobilisation has been studied extensively in the lower limb^33,34^ and has been associated with decreased pain scores, and rapid recovery of muscle function.^35–38^ Early research following rotator cuff repair suggests integrity of repair is maintained when comparing early versus delayed post-operative mobilisation.^39^ The possibility of earlier safe mobilisation has important implications for the wide range of patients with TASD, for example, poor compliance with immobilisation periods,^40^ working populations and loss of earnings,^41^ the psychological impact of not being engaged in leisure and sport,^42^ and for older patients, the risk of stiffness due to prolonged immobilisation.^43^

Seventy six percent of participants in the studies included in this review had arthroscopic stabilisation, designed to minimise recovery times.^44^ Small incisions and less invasive techniques, require less hospitalisation time, allowing patients to ideally return to their former activities sooner than open surgery. All studies in our review involved a Bankart in which the torn anterior labarum and anterior band of the inferior glenohumeral ligament is reattached to the glenoid rim.^45^ Other techniques such as Latarjet and Remplissage procedures, are increasingly common and involve bony and cartilaginous structures, for which physiological healing times will vary. Variation in types of stabilisation surgery needs to be considered for future rehabilitation programmes.

### Biopsychosocial approach

Shoulder stabilisation surgery requires many months of rehabilitation to achieve a successful outcome. Patient expectation of recovery, pain, self-efficacy and fear avoidance predict outcome following surgery and rehabilitation.^46–50^ The fundamental components for all treatment programmes in this review included optimising ROM, strength and functional recovery. None stated consideration of the psychological or social factors that may influence, for example return to play. When an athlete sustains an injury, it has psychosocial impacts^51–53^ and many athletes do not return to sport due to fear of re-injury.^54^ A common misconception historically is that physical and psychosocial recovery occurs at the same time.^55^

Several systematic reviews have explored the role of preoperative self-efficacy and expectations of recovery in patients with chronic shoulder pain.^49,56^ One of these reviews highlighted that the high levels of resilience and preoperative expectations are significantly associated with low levels of post-operative pain intensity.^49^ Similarly, high levels of depressive symptoms anxiety, pain catastrophising, emotional distress and somatisation are significantly associated with high levels of pain intensity.^49^ These finding are in keeping with those of Henn et al.^46^ in that greater preoperative expectations correlated with better post-operative performance on the simple shoulder test, Disabilities of the Arm, Shoulder and Hand scale and the Visual Analogue Scale following rotator cuff repair.^46^ We therefore recommend pre-rehabilitation based on an integrated biopsychosocial model.

We recommend combining clinical measures and PROMs designed to capture impairment, function and participation following surgery and post-operative rehabilitation. We recommend PROMs, tested for their validity, reliability and responsiveness in capturing change specific to shoulder instability. Examples might include the Western Ontario Shoulder Index (WOSI) and Oxford Shoulder Instability.^57^ Return to play is an important measure for many people^58^ and should be clearly defined.

### Strengths and limitations

The PRISMA guideline extension for scoping reviews^23^ was strictly followed throughout this review. Discussions with research and clinical experts at each stage of the review contributed to the search strategy, eligibility criteria, data extraction, and defining categories to subgroup each intervention component. The main limitation of this review is that only 2 clinical studies compared rehabilitation programmes, one of which was not randomised.

## Conclusion

Following TASD, there is considerable variability in the post-operative immobilisation period and when each type of exercise is introduced. There is a lack of research comparing the effect of different rehabilitation programmes and a lack of evidence to guide post-operative rehabilitation. There is no consensus on the definition of accelerated rehabilitation, or recommended outcome measures. Recent advancements in surgical procedures and varying populations presenting with TASD may account in part, for some of the variability. We recommend incorporating a biopsychosocial approach alongside traditional biomechanically based interventions. Clinical consensus of standardised terminology and stages of rehabilitation is required prior to developing a randomised controlled trial.
